# Everyday Discrimination in Young Adulthood and Depressive Symptoms at Early Midlife: The Moderating Role of Parent–Child Relationships

**DOI:** 10.3390/ijerph22091323

**Published:** 2025-08-26

**Authors:** Binoli Herath, Xing Zhang

**Affiliations:** College of Health Solutions, Arizona State University, 425 N. 5th Street, Phoenix, AZ 85004, USA

**Keywords:** everyday discrimination, young adulthood, depressive symptoms, parent–child relationships, early midlife

## Abstract

Discrimination has been linked to greater stress and higher levels of depressive symptoms. However, there has been no research to date that has examined how life course inequality due to everyday discrimination is associated with mental health outcomes later in life. Using data from Waves I, IV, and V of the National Longitudinal Study of Adolescent to Adult Health (Add Health) from 1994 to 2018, we examined how everyday discrimination in young adulthood (Wave IV) was associated with depressive symptoms at early midlife (Wave V). We also examined how parent–child relationships in young adulthood (maternal and paternal closeness; satisfaction of communication with mothers and fathers) moderated this association. We analyzed two sub samples: a mother sample (n = 9390) and a father sample (n = 8229). Results from both showed that everyday discrimination in young adulthood was significantly associated with depressive symptoms at early midlife, and parent–child relationships served as significant protective factors against depression. Mental health policy and intervention efforts should address how discrimination experienced in young adulthood can have enduring adverse effects on mental health into early midlife and invest in strategies that promote supportive parent–child relationships as protective resources.

## 1. Introduction

Discrimination is a socially structured and sanctioned phenomenon, driven by ideology and manifested through interpersonal interactions, intended to promote privileges for a dominant group while depriving others of those privileges [1]. Experiencing discrimination is believed to provoke stress and trauma responses, which may contribute to adversity over the life course through the development of mental health disorders [2,3]. Discrimination has been linked to negative psychological effects, including depression [4,5]. A widely referenced explanation for how discrimination may contribute to negative health outcomes is the biopsychosocial model [6]. According to this model, experiencing discrimination can trigger a prolonged physiological stress response, resulting in biological and psychological dysfunction (e.g., immune and inflammatory responses, heightened allostatic load, and mental health symptoms), which subsequently influences long-term morbidity [6].

A growing body of evidence demonstrates a strong correlation between the experiences of discrimination and poor mental health across various racial and ethnic groups [1,7,8]. African Americans are disproportionately affected by discrimination, stemming from both their racial minority status and the historical and ongoing racial injustices in the United States. Studies consistently indicate that Black individuals experience higher rates of depression and depressive symptoms compared to non-Hispanic White Americans. Racial discrimination has been recognized as a powerful stressor associated with higher levels of depressive symptoms [9,10] and an increased risk of depression [11,12]. While early sociological research acknowledged that racial health disparities are largely driven by social determinants [13,14], contemporary studies reinforce these findings, emphasizing racism, more specifically racism-related stress, as a fundamental cause of racial differences in health outcomes [15,16,17]. For these reasons, discrimination can be recognized as a significant social determinant of health that may contribute to health disparities.

Everyday discrimination refers to the frequent unfair treatment, subtle insults, and other forms of mistreatment that privileged groups direct toward disadvantaged groups [18]. These discriminatory experiences encompass various forms of interpersonal mistreatment, such as being treated with less courtesy or respect, receiving poorer service in public places, or being perceived as unintelligent or dishonest [19]. This perceived form of discrimination differs from actual or external indicators of discrimination because it is based on individual self-assessment rather than objectively defined measures [20,21]. As a result, it serves as an indirect measure influenced by emotions and cognition. However, subjective experiences play a crucial role in defining discrimination, as perceptions of unfair treatment can vary between individuals.

The frequency of perceived discrimination also plays a key role in making it a stressful experience. Williams and Mohammed (2009) suggest that frequently encountering discrimination can create constant pressures or ongoing threats, leading to increased distress [21]. Everyday discrimination captures the regularity of such experiences. Repeated exposure to discrimination is increasingly recognized as a major source of stress [12,17,22,23]. To better understand these experiences, the Everyday Discrimination Scale (EDS) [17] serves as a foundational scale in numerous studies examining various forms of discrimination. Researchers have frequently used this scale to assess multiple forms of discrimination, including racial, ethnic, gender-based, and age-related discrimination, as well as more general experiences within broader populations [24,25].

Findings from studies utilizing the EDS indicate that perceptions of discrimination remain prevalent in the United States and that different social groups report varying levels of exposure to discrimination [19]. Greater scores on the EDS are associated with an increased risk of poor health and well-being [7,21]. The most frequently documented link between perceived discrimination and health pertains to mental health outcomes, particularly psychological distress and depressive symptoms [12,26,27]. Evidence from longitudinal research strongly supports the idea that experiences of discrimination over time contribute to the development of depression. Studies have found that individuals who reported discrimination experienced higher levels of depressive symptoms in later years compared to those who did not [28,29]. Similar patterns have been observed among a sample of African American adolescents in schools in Baltimore, showing that discrimination was positively associated with depressive symptoms one year later across all waves of measurement [30]. Likewise, Schulz et al. (2006) found that among African American women in the Detroit metropolitan area, an increase in discrimination over time was associated with a rise in depressive symptoms [31]. Collectively, these findings suggest that repeated exposure to discrimination plays a significant role in the onset and progression of depression.

### 1.1. The Life Course, Discrimination, and Depressive Symptoms

Longitudinal studies on life chances, health, and well-being emphasize the necessity of adopting a life course perspective when examining life course inequality, incorporating data spanning decades [32,33,34]. While some risk factors have immediate effects, others take time to emerge. Early life risk factors not only lead to future health outcomes but also contribute to long-term cumulative effects. The accumulation of these risk factors over the life course contributes to the variability in health outcomes later in life [35,36]. This effect can be understood through the lens of cumulative disadvantage theory, which explains how the interplay of biological, behavioral, and social factors shapes health outcomes over time [37,38]. A fundamental principle of this theory is that early life advantages or disadvantages influence exposure to health-compromising risk factors, such as adverse life events. Factors including race/ethnicity, socioeconomic status (SES), and gender shape individuals’ vulnerability to social, behavioral, and biological risks from birth onward [39]. Cumulative disadvantage can be understood as the accumulation of social, economic, and personal stressors resulting from unequal access to resources and opportunities, which in turn heightens biological vulnerability to disease [40]. Social inequalities become biologically embedded through prolonged physiological stress responses, leading to increased susceptibility to illness and functional decline [38]. In this way, chronic stress serves as a key mechanism driving health disparities.

Experiences of discrimination occur both at institutional/systemic and individual levels [41]. Everyday discrimination has a more chronic and ongoing nature, and therefore, it gradually accumulates over time, contributing to cumulative disadvantage and increasing the risk of depression [42]. These repeated stressors trigger chronic physiological responses, such as heightened cortisol levels and systemic inflammation, which are linked to mental health deterioration [43]. The persistent stress from everyday discrimination could lead to emotional exhaustion, feelings of helplessness, and internalized stigma, all of which reinforce depressive symptoms, or feelings of sadness, loneliness, and loss of interest in daily activities [44]. Discrimination restricts access to social and economic resources by limiting educational and employment opportunities, reducing financial stability, and eroding social support systems, further exacerbating psychological distress [45]. Over time, these disadvantages compound, making individuals more vulnerable to depression. The cumulative impact of everyday discrimination illustrates how seemingly minor but frequent negative interactions can create long-term psychological burdens. This sustained exposure over time can contribute to cumulative disadvantage, ultimately resulting in more severe mental health challenges, including greater levels of depressive symptoms.

### 1.2. Moderator Role of Parent–Child Relationships

Social relationships significantly influence health outcomes as a key social determinant of health [46]. Individuals who experience greater social isolation and fewer interpersonal interactions are at increased risk of poorer mental health [47,48,49]. Social support plays a crucial role in shaping mental health outcomes, particularly in mitigating the adverse effects of discrimination. It can take the form of emotional support, including positive interactions, empathy, encouragement to express feelings, or informational support, such as providing advice, guidance, or feedback [46]. Recent research has shown that individuals who experience racial discrimination but have access to social support are less likely to suffer adverse mental health outcomes [9,46,50,51]. Social support has been shown to protect against various mental health challenges, including depression [52,53,54].

Although the precise mechanisms are not yet fully understood, the existing literature suggests two possible explanations for how social support might serve as a protective factor against depression [46,50]. First, studies have shown that social support functions as a buffer in the relationship between discrimination and depressive symptoms by offering emotional and informational resources that help individuals navigate negative experiences effectively [46,50]. Second, social support also functions as a key resource for coping with stress [55]. A growing body of research highlights its stress-buffering effects, particularly in mitigating the negative impact of discrimination on mental health [45,50]. Studies suggest that discrimination contributes to depression through heightened stress levels, but strong social support systems can help alleviate these effects [20]. In a review examining self-reported discrimination and health, Lewis et al., (2015) identified social and emotional support as a promising protective factor that could buffer against discrimination’s negative health impacts [20].

Support from family fosters a sense of belonging, which reduces stress and, in turn, lowers the likelihood of depression [56,57]. This highlights the importance of maintaining strong social connections as a protective mechanism against psychological distress. Existing studies highlight the protective effects of social support from family against psychological distress [58,59]. For instance, studies have indicated that African Americans who receive frequent emotional support and maintain regular contact with extended family members are less likely to meet the criteria for depression [58,59]. Among older African Americans, social support has been identified as a critical factor in reducing depression, depressive symptoms, and psychological distress [52]. Furthermore, research suggests that family support predicts higher levels of life satisfaction, happiness, and self-esteem among African Americans [60].

Within a family, strong parent–child relationships, characterized by maternal and paternal closeness and satisfaction with communication, provide a foundation for emotional and psychological well-being by enabling children to share their experiences with discrimination and receive guidance, reassurance, and validation from their parents [61,62]. This support can serve as a protective factor against the harmful effects of discrimination by fostering resilience and reducing emotional distress. Drawing from attachment theory [63], theories on the balance between connectedness and individuation [64], and theories regarding parenting styles [65] it is suggested that children who have close, supportive relationships with their parents experience better developmental outcomes and fewer depressive symptoms. In contrast, problems in parent–child relationships have consistently been identified as a risk factor for developing depressive symptoms. Poor-quality parent–child relationships may increase depressive symptoms, as children in these relationships typically receive less emotional support during stressful times [66]. Feeling accepted and valued by parents is believed to enhance individual self-esteem and self-efficacy, providing resilience against depressive emotions [67].

Longitudinal studies have examined the effects of parent–child relationship quality and depressive symptoms [68,69]. The majority of these studies have been performed among adolescents, where higher parental support was associated with decreased depressive symptoms in girls, but this effect was not observed in boys [67]. Adolescents who perceived their relationship with their mother as high-quality reported fewer depressive symptoms one year later [70]. Additionally, both maternal and paternal support were found to reduce depressive symptoms during late adolescence [71]. Several studies have examined how interactions and communication between parents and children can influence the development of depression [72]. For example, research has found that in young children experiencing depression, there was significantly less communication with their mothers, both in the quantity and depth [72]. Mothers of depressed children also saw their relationships as more distant and less affectionate compared to mothers whose children were not depressed [72].

While research on parent–child relationships during the shift from adolescence to young adulthood remains limited, existing evidence suggests that earlier patterns of interaction with parents often persist as children mature and enter new life stages [73,74]. Early bonds between parents and children lay the foundation for intergenerational relationships later on [75]. Prior studies have found that young adults’ reports of family cohesion, parental warmth, and emotional closeness in adolescence are positively linked to their current feelings of closeness with their parents [76]. As children assume adult roles, such as completing their education, marrying, cohabiting, securing full-time employment, or becoming parents themselves, these transitions can reshape their relationships with their parents [73,74]. For instance, Thornton et al., (1995) observed that affective closeness between parents and children often increases as young people move into adulthood, due to shared life experiences [76]. Over time, this bond evolves from early dependence to a more reciprocal relationship marked by mutual understanding and support [77]. Longitudinal studies, such as the National Longitudinal Study of Adolescent to Adult Health (Add Health), offer valuable insights into the continuity and transformation of parent–child relationships during this developmental period.

### 1.3. Purpose of the Present Study

**Figure 1:**
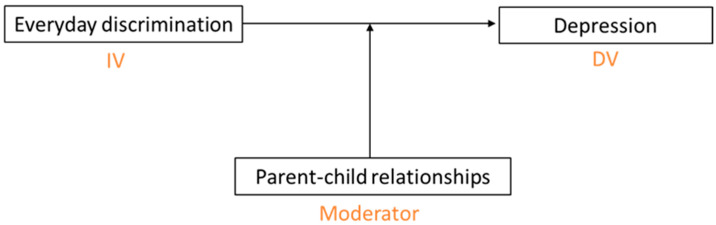
Conceptual framework illustrating the moderating effect of parent–child relationships on the association between everyday discrimination and depression. IV – Independent variable; DV – Dependent variable.

This study uses Waves I, IV, and V of the National Longitudinal Study of Adolescent to Adult Health (Add Health) to investigate the relationship between everyday discrimination and depressive symptoms [78]. The main goals of this study are to examine (1) whether there is an association between everyday discrimination in young adulthood and depressive symptoms in early midlife and (2) how this relationship is moderated by parent–child relationships in young adulthood. We hypothesized that there would be a positive association between everyday discrimination and depressive symptoms (Hypothesis 1). We also hypothesized that the association would be modified by parent–child relationships, such that the depressive symptoms would be exacerbated for those adults who were less connected with their parents (Hypothesis 2). For young adults who were closer and had more satisfying communication with their parents, the relationship between discrimination and depression would be lessened or buffered. Our conceptual framework is depicted in Figure 1.

Building on the framework depicted in Figure 1, we anticipate that parent–child relationships will moderate the association between everyday discrimination in young adulthood and depressive symptoms at early midlife. Specifically, young adults who report stronger emotional bonds and greater satisfaction with communication with their parents are expected to exhibit lower levels of depressive symptoms as they transition into early midlife. By fostering a supportive environment, these relationships may help mitigate depressive symptoms associated with everyday discrimination, reinforcing the importance of social support networks in long-term mental health.

## 2. Materials and Methods

### 2.1. Data

We used data from the National Longitudinal Study of Adolescent to Adult Health (Add Health; https://addhealth.cpc.unc.edu/; accessed on 25 August 2025), a nationally representative, prospective cohort of adolescents who were in middle or high school during the 1994–1995 academic year [78]. Comprehensive details regarding data collection can be found on the Add Health website and in previous publications [79]. In summary, Add Health is a nationally representative longitudinal cohort study that began in 1994–1995 (Wave I; ages 12–21) with over 20,000 adolescents in the 7th–12th grades across the United States. The study employed a school-based sampling strategy, selecting 80 high schools along with their corresponding feeder schools from a comprehensive list of U.S. high schools in 1994. Participants have been followed up in 1995–1996 (Wave II; ages 13–22), 2001–2002 (Wave III; ages 18–26), 2008–2009 (Wave IV; ages 24–32), and 2016–2018 (Wave V; ages 32–42) respectively from adolescence into early midlife through five waves of in-home interviews. For this present study, we used data from Waves I, IV, and V.

### 2.2. Measures

#### 2.2.1. Depressive Symptoms

At Wave V, participants were asked to complete the short-form version of the Center for Epidemiologic Studies Depression Scale (CES-D-5) [44]. Participants were asked to indicate how often they experienced feelings in the provided statements over the past 7 days: (1) “I felt that I could not shake off the blues, even with help from my family and friends;” (2) “I felt depressed;” (3) “I was happy;” (4) “I felt sad;” and (5) “I felt that life was not worth living.” Responses were coded categorically, and ranged between 0 (never or rarely) and 3 (most of the time or all of the time). Responses to all 5 items were summed (α = 0.82), such that higher scores indicated more depressive symptoms.

#### 2.2.2. Everyday Discrimination

The original Everyday Discrimination Scale (EDS) [17], is a nine-item measure in which respondents were asked to report how often they experienced discrimination in their everyday lives. During the Wave IV interviews, a single-item measure was used to assess perceived discrimination. This item combines the first two items of the EDS; “In your day-to-day life, how often do you feel you have been treated with less respect or courtesy than other people?” Participants provided ratings ranging from “never” (0) to “often” (3). Due to data limitations, the Add Health data did not have the full EDS. This shortened measure that combines two items from the EDS has demonstrated strong construct and criterion validity compared to the original nine-item scale. For example, the correlations between the two specific items of being treated with “less respect” and “less courtesy” appeared to be strong in prior work by Benner and colleagues in 2024 [80]. Similar to the original EDS, a follow-up question was posed to participants who reported experiencing discrimination “sometimes” (2) or “often” (3) to indicate the reason they believe they were discriminated against (e.g., attribution tied to race, gender, skin color, weight, etc.).

#### 2.2.3. Parent–Child Relationships

We used two measures of parent–child relationships as moderators: parental closeness and satisfaction with communication with parents. At Wave IV, separate questions on closeness were posed to each parent: “How close do you feel to your (mother figure)?” and “How close do you feel to your (father figure)?” Participants provided ratings ranging from “not at all close” (1) to “very close” (5). Participants also rated their satisfaction with communication with each parent using the items “You are satisfied with the way your (mother figure) and you communicate with each other” and “You are satisfied with the way your (father figure) and you communicate with each other” on a scale from 1 (“strongly agree”) to 5 (“strongly disagree”).

#### 2.2.4. Covariates

We accounted for covariates, such as race/ethnicity, age, gender, immigrant generation status, marital status, employment status, household income level, highest educational attainment, family structure, and CES-D scores at Wave I. The list of variables and their data source (Wave from Add Health) used in the analyses are presented in Table 1.

### 2.3. Analytic Sample

We limited our analysis to four major racial and ethnic groups in the sample: non-Hispanic White, non-Hispanic Black, non-Hispanic Asian, and Hispanic. Of the 20,745 respondents who completed Wave I of Add Health, only those who participated in Waves I, IV, and V and had valid sampling weights at Wave V were included in our sample (n = 10,912). Because we drew data from three waves of the survey, there were missing responses on some measures. Respondents were missing data for variables, including gender (n = 1), race/ethnicity (n = 127), year of birth at Wave I (n = 6) (therefore, age could not be determined at Wave IV (n = 6), immigrant generation status (n = 1), employment at Wave IV (n = 4), marital status at Wave IV (n = 12), household income level at Wave IV (n = 625), highest educational attainment at Wave IV (n = 4), CES-D score at Wave I (n = 7), CES-D score at Wave V (n = 15), closeness to mother figures (n = 620), closeness to father figures (n = 1906), satisfaction of communication with mothers (n = 623), satisfaction of communication with fathers (n = 1911), perceived less respect or courtesy at Wave IV (n = 2), and missing region data (n = 228). Respondents with missing data were excluded, except for those missing data on both closeness and satisfaction of communication with mother and father figures. Given that there was more missing data on relationships with fathers than relationships with mothers, we prepared two separate analytic samples in accordance with previous work [81]: one including participants who responded to the question on closeness to their mother figures (n = 9390), referred to hereafter as the *mother sample*, and the other including participants who responded to the question on closeness to their father figures (n = 8229), referred to hereafter as the *father sample*.

### 2.4. Analytic Strategy

We began by generating descriptive statistics for respondents in the mother and father samples using Stata 18.0. To examine the association between everyday discrimination in young adulthood and depressive symptoms in early midlife, we conducted ordinary least squares (OLS) regression analyses. We then tested whether maternal and paternal closeness moderated this association by including interaction terms between everyday discrimination and closeness to each parent in separate OLS models. A similar approach was used to assess the moderating role of satisfaction with communication with each parent. All regression models were controlled for the covariates described earlier in the methods section. In accordance with the Add Health user guidelines [82], we applied Wave V cross-sectional weights to ensure national representativeness of adolescents who were in the 7th–12th grades in the U.S. during the 1994–1995 school year.

## 3. Results

Summary statistics for the two analytic samples are presented in Table 2. The mother sample included participants who responded to the question on closeness to their mother figure (n = 9390), whereas the father sample included participants who responded to the question on closeness to their father figure (n = 8229). At Wave IV, the mean age of both samples was 28.8 years old. Half of the sample (50%) were women, and nearly 70–73% were non-Hispanic White. A significant majority of the sample was born in the U.S., and the family characteristics revealed that 58–63% of the participants were living with their biological parents at Wave I. At Wave IV, the largest group of respondents (43%) reported they had received some college or vocational/technical training, and most (66%) were employed for at least 10 h/week. Common reasons that participants believed they were discriminated against included race, age, education/income, and other factors. However, as there were many missing responses for this question, the reasons for discrimination were excluded from the OLS analysis.

### 3.1. Everyday Discrimination and Depression

Table 3 shows the OLS regression analysis conducted to check the association between everyday discrimination in young adulthood and depressive symptoms at early midlife for each of the mother and father samples, respectively. We conducted a regression model first without controls and then included controls. Everyday discrimination in young adulthood was positively associated with depressive symptoms (B = 0.13, *p* < 0.001) at early midlife in both analytic samples, supporting our Hypothesis 1, and this association remained significant even after adjusting for covariates (B = 0.10, *p* < 0.001).

Non-Hispanic Asians in the mother sample had significantly fewer depressive symptoms relative to Non-Hispanic Whites (B = −0.07, *p* < 0.05). Furthermore, married individuals in both samples reported significantly lower depressive symptoms relative to unmarried individuals (B = −0.06, *p* < 0.001). Individuals who completed a college degree or above had significantly fewer depressive symptoms relative to those who completed less than a high school degree (*p* < 0.001). Similarly, individuals who lived in a middle- to high-income household (*p* < 0.001) had significantly lower levels of depression compared to those who lived in a low-income household setting. In contrast, adolescents who were raised with a single parent had significantly higher depressive symptoms in early midlife than those who were raised with two biological parents (B = 0.04, *p* < 0.05). Moreover, adolescents who had reported depressive symptoms (CES-D) at Wave I were more likely to report depression in early midlife (B = 0.16, *p* < 0.001).

### 3.2. Parent–Child Relationships as a Moderator

#### 3.2.1. Parental Closeness

We ran separate regression models with maternal closeness (mother sample) and paternal closeness (father sample) as moderators in Table 4. Results indicate that the association between everyday discrimination in young adulthood and depressive symptoms at early midlife were still significant in both samples. Maternal closeness (B = −0.04, *p* < 0.05) and paternal closeness (B = −0.03, *p* < 0.05) were found to be significant protective factors against depression. However, parental closeness did not serve as a significant moderator for this relationship. Moreover, being Non-Hispanic Asian (*p* < 0.05), being married (*p* < 0.001), having a middle- to high-household income level (*p* < 0.001), and completing college education or above (*p* < 0.001) were also additional protective factors for depression. In contrast, depressive symptoms (CES-D) in adolescence were a significant risk factor (*p* < 0.001) for depression in early midlife.

#### 3.2.2. Satisfaction with Communication with Parents

Similarly, we ran separate regression models for satisfaction with communication with mothers (mother sample) and fathers (father sample) as moderators, as indicated in Table 5. However, the results indicate that the association between everyday discrimination and depressive symptoms were significant only for the mother sample (B = 0.13, *p* < 0.05). Satisfaction with communication with mothers and fathers (B = −0.03, *p* < 0.05) were found to be significant protective factors for depression. However, satisfaction with communication with parents did not significantly moderate the relationship between everyday discrimination and depression.

## 4. Discussion

Everyday discrimination has been identified as a significant risk factor contributing to adverse mental health outcomes. We used a large dataset from the National Longitudinal Study of Adolescent to Adult Health (Add Health), to examine how everyday discrimination in young adulthood influenced depressive symptoms at early midlife and how parent–child relationships potentially moderated this association. Our results indicate that everyday discrimination in young adulthood was strongly associated with depressive symptoms in early midlife, supporting Hypothesis 1. We used parental closeness and satisfaction with communication with parents as moderators. Closeness to parents and satisfaction with communication with each parent were found to be significant protective factors against depression. However, neither served as significant moderators for this relationship. In contrast to our Hypothesis 2, we found that parent–child relationships did not moderate the association between everyday discrimination and depressive symptoms. We also found some other protective factors for depressive symptoms, such as being Non-Hispanic Asian, being married, completing a college degree or more, and living in a middle- to high-income household. Moreover, being raised by a single parent and having depressive symptoms in adolescence were identified as risk factors for depressive symptoms at early midlife.

Everyday discrimination is a perceived form of discrimination based on self-assessment or subjective experience, and it can vary between individuals [20]. A person might feel they were less respected in the same situation, where another person might not feel that way. Whether someone interprets an experience as discriminatory or not can vary [83]. Moreover, there is a possibility of reverse causality, where individuals with depression (at baseline) might be more sensitive to treatment with less respect or courtesy than others. This could result in inter-individual variability in subjective accounts of discrimination. Common reasons that participants believed they were experiencing everyday discrimination were race, age, and education/income. However, due to data limitations, we were unable to analyze how these different types of discrimination were associated with developing depressive symptoms in later life.

Furthermore, in this study, we found some significant predictors from young adulthood or earlier that were associated with depressive symptoms at early midlife. These factors are wider social determinants, such as race, family structure, marital status, household income level, and level of education, which shape individuals’ likelihood of experiencing depressive symptoms. For instance, educational attainment can serve as a supportive factor against depression, as individuals with a college degree are more likely than others to have more resources that better enable them to adopt protective factors [16] or to have better coping strategies and social support that can buffer the stress from discrimination [84]. Therefore, it is evident that factors such as educational status, economic stability, and level of social support may contribute to vulnerability to discrimination. Although research shows that women are more likely to experience depression than men due to gender discrimination, this was not reflected in this analysis. Our results are comparable with the study by Wang and Narcisse (2025), which was a cross-sectional study using data from the National Health Interview Survey, which includes a nationally representative sample of U.S. adults where associations between discrimination and screening positive for depression and anxiety varied by race and ethnicity but not by sex [85].

Everyday discrimination illustrates how seemingly minor but frequent negative interactions can create long-term psychological burdens. As this data was drawn from a longitudinal study, it can be used to examine life course determinants of health, as some risk factors take time to emerge. Scholars who studied the life course relationship between racial discrimination and depressive symptoms have proposed two mechanisms that may explain this association. One explanation suggests that discrimination limits opportunities for certain groups [4,86]. The other perspective highlights that the psychological and physiological stress responses triggered by racial discrimination can accumulate over time and contribute to deteriorating health [8,12,17]. When experienced early in life and reinforced across different social contexts, this can create cumulative disadvantages by restricting access to social/economic resources, limiting educational/employment opportunities, affecting financial stability, socioeconomic mobility, and increasing stress exposure [17]. This can further exacerbate psychological distress. From a life course perspective, additional risk factors, such as experiencing depressive symptoms during adolescence, may interact with everyday discrimination in young adulthood to create a cumulative disadvantage. These multiple exposures across developmental stages increase vulnerability and elevate the likelihood of depressive symptoms by early midlife. Therefore, the cumulative and persistent nature of discrimination plays a crucial role in shaping long-term health trajectories.

In this context, social support becomes especially important. The closest sources of support are often family members, particularly parents. In this study, we observed that parent–child relationships were important protective factors against depression. This aligns with previous research demonstrating that positive and supportive parent–child relationships serve as a protective factor against depression by reducing the risk of developing depressive symptoms [68,69]. Such relationships, marked by warmth, security, and open communication, help buffer the effects of stress and adversity, enabling individuals to better cope with challenges and foster mental well-being. Parent–child relationships endure across the lifespan, and the emotional quality of these relationships is especially important, as strong, affectionate bonds benefit both generations and are linked to lower rates of depression. These relationships are inherently reciprocal and contingent, shaped by mutual influence and support. 

A considerable body of research focuses on family relationships/supportive parenting practices as a potential source of protection to counter the impact of discrimination on depressive symptomatology [9,58,87,88]. A longitudinal study by Brody and colleagues (2006) found that perceived discrimination in adolescence was associated with increased depressive symptoms, but this association was weaker for youth who received parental warmth and support [9]. Multi-group analyses revealed that nurturant-involved parenting was moderating this relationship (*p* < 0.01) [9]. In our study, we did not find that parent–child relationships moderated the impact of everyday discrimination on depressive symptoms, even though strong parental relationships were independently associated with lower depressive symptoms. A possible explanation is that the protective role of parent–child relationships may be confounded by other sources of social support that become particularly prominent in young adulthood. At Wave IV of Add Health, participants were aged 24–32 years, a period often characterized by life transitions, such as leaving the parental home, forming social connections, or marrying. During this stage, peers, spouses, or romantic partners may play a more central role in providing social support, which we did not account for in our analyses. Consistent with this possibility, our findings show that being married was a significant protective factor against depression, and this association remained robust even after including moderator variables in the regression model, signaling potential confounding effects. Including these additional sources of support was beyond the scope of the current study, but they may have attenuated the moderating influence of parent–child relationships.

Developmental psychology research demonstrates that supportive parent–child relationships are critical for positive mental health outcomes and help reduce the risk of depressive symptoms in children [89]. Bowlby’s attachment theory [63] highlights that secure attachment with parents provides a “secure base” that fosters self-esteem, emotional regulation, academic success, and healthy social relationships across the life course. Children who develop secure relationships with their parents are therefore less vulnerable to depression [90], which may explain why parent–child relationships moderate the association between discrimination and depression during childhood and adolescence. In adulthood, however, individuals increasingly rely on peers, partners, and extra-familial social networks for support, diminishing the relative importance of parent–child relationships. This shift may be due to a saturation effect, where the need for parental support decreases as individuals develop other close relationships that fulfill similar needs. As a result, parental or family-based interventions may be less effective in mitigating discrimination-related depressive symptoms in young adults or older adults, reiterating the importance of implementing such interventions earlier in life.

## 5. Limitations

Though this study illuminated the relationship between everyday discrimination, parent–child relationships, and depressive symptoms, this study was not free from limitations. First, due to data limitations from the Add Health survey, the Everyday Discrimination Scale (EDS) at Wave IV of Add Health only included one measure that combined two items from the EDS, limiting the ability to capture the full complexity and comprehensiveness of perceived discrimination. Second, the Everyday Discrimination Scale was not used in earlier waves of Add Health, so we could not include it as a covariate to control for respondents who may have experienced feeling treated with less respect or courtesy than others earlier in their lives. Third, this study was not causal in nature, but associational. Therefore, we were unable to establish a cause–effect relationship between everyday discrimination and depressive symptoms. Fourth, we used self-reported measures of depressive symptoms from the CES-D-5, rather than formally or clinically diagnosed measures of depression. Fifth, we did not examine respondents’ ways of coping or social support (outside of parents) that respondents received, because the study was focused on the moderation effect of parent–child relationships only. Social support outside of the family might have confounded this hypothesized moderating relationship in several ways. Social support from other sources (e.g., friends, school, community) could have direct effects on the outcome (depressive symptoms), independent of the parent–child relationship. On the other hand, the effect of the parent–child relationship might be dependent on the level of social support from other sources. Therefore, excluding extra-familial support from the analysis may over- or underestimate the true strength of the parent–child relationship’s moderating effect. Sixth, we did not examine the specific types of discrimination faced and their associations with depressive symptoms due to data limitations in Add Health. Only a subset of respondents were asked why they experienced a certain type of discrimination if they responded “sometimes” or “often” to experiencing being treated with less respect and courtesy than other people. Thus, we were unable to differentiate whether the different types of discrimination experienced led to variation in depressive symptoms. Lastly, while prior research has documented variation in intergenerational solidarity by race, ethnicity, gender, socioeconomic status, and across the life course [74], our study did not examine these differences.

## 6. Conclusions

In conclusion, in our study, we found that everyday discrimination in young adulthood was associated with increased depressive symptoms in early midlife, confirming cumulative disadvantage theory [37]. Furthermore, this study highlights parent–child relationships as an important source of support for mental health in the transition from young adulthood to early midlife. Future research can explore whether and how different types of discrimination faced (such as that based on gender, race/ethnicity, and socioeconomic status) shape depressive symptoms, use causal approaches, such as experimental approaches and discontinuity designs, and identify other potential moderating factors in the relationship between everyday discrimination and mental health, such as educational attainment, income, and other forms of socioeconomic status. Future research can also use datasets that include the full nine-item Everyday Discrimination Scale, such as the National Survey of American Life [80].

## Figures and Tables

**Table 1:** Description of variables used in the analysis.

Variable Type	Variables	Data Source (Wave from Add Health)
Dependent variable	Depression (CES-D-5)	Wave V
Independent variable	Everyday discrimination (EDS)	Wave IV
Moderator	Parent-child relationships	Wave IV
Covariates	Race/ethnicity	Wave I
	Age	Wave IV
	Gender	Wave I
	Immigrant generation status	Wave I
	Marital status	Wave IV
	Employment status	Wave IV
	Household income level	Wave IV
	Highest educational attainment	Wave IV
	Family structure (living with parents or not)	Wave I
	Depression (CES-D-5)	Wave I

**Table 2:** Weighted descriptive statistics for the analytic sample.

Variable	Mother Sample (N = 9390)	Father Sample (N = 8229)
Gender		
Male	0.50	0.50
Female	0.50	0.50
Age at Wave IV, mean (SD)	28.88 (0.11)	28.86 (0.12)
Race/ethnicity		
Non-Hispanic White	0.70	0.73
Non-Hispanic Black	0.14	0.12
Hispanic	0.12	0.11
Non-Hispanic Asian	0.04	0.03
Immigrant generation status		
Born in the U.S.	0.94	0.94
Foreign born	0.05	0.05
Marital status		
Never married	0.47	0.46
Married	0.53	0.54
Employment status		
Not employed for at least 10 h/week	0.33	0.34
Employed for at least 10 h/week	0.66	0.66
Household income level		
USD 24,999 and less	0.16	0.15
USD 25,000–49,000	0.29	0.28
USD 50,000 or above	0.55	0.57
Highest educational attainment		
High school or less	0.22	0.22
Some college or vocational	0.43	0.43
Completed college or above	0.34	0.36
Family structure		
Two biological parents	0.58	0.63
Two parents	0.16	0.16
Single parent	0.21	0.16
Other	0.04	0.03
Depression (CES-D) at Wave I, mean (SD)	0.48 (0.01)	0.47 (0.01)
Reason for everyday discrimination	(n = 2110)	(n = 1799)
Ancestry or national origin	0.02	0.02
Gender	0.05	0.05
Race	0.08	0.07
Age	0.09	0.10
Religion	0.01	0.01
Height/weight	0.05	0.05
Skin color	0.01	0.01
Sexual orientation	0.00	0.00
Education/income	0.09	0.09
A physical disability	0.02	0.03
Other	0.59	0.59

**Table 3:** Summary of OLS regression analysis for variables predicting depression.

Variable	Mother Sample		Father Sample	
	B	SE	B	SE
Everyday discrimination	0.13 ***	0.01	0.13 ***	0.01
Regression including covariates	0.10 ***	0.01	0.10 ***	0.01
Gender	0.01	0.01	0.02	0.02
Race				
Non-Hispanic Black	−0.01	0.03	0.01	0.03
Hispanic	−0.02	0.02	−0.01	0.03
Non-Hispanic Asian	−0.07 *	0.03	−0.07	0.04
Age	0.00	0.00	0.00	0.01
Family structure				
Two parents	0.01	0.02	0.01	0.02
Single parent	0.04 *	0.02	0.02	0.02
Other	0.06	0.04	0.09	0.05
Immigrant generation status	0.02	0.04	0.04	0.04
Marital status	−0.06 ***	0.01	−0.06 ***	0.02
Employment status	−0.03	0.01	−0.02	0.01
Household income level				
USD 25,000–49,000	−0.05 *	0.02	−0.09 **	0.03
USD 50,000 or above	−0.09 ***	0.02	−0.13 ***	0.03
Highest educational attainment				
Some college or vocational	−0.01	0.02	−0.01	0.02
Completed college or above	−0.10 ***	0.02	−0.09 ***	0.02
Depression (CES-D) at Wave I	0.16 ***	0.02	0.16 ***	0.02

*—*p* < 0.05; **—*p* < 0.01; ***—*p* < 0.001. Note—all covariates included.

**Table 4:** Summary of OLS regression analysis for everyday discrimination and depression, with maternal closeness and paternal closeness as moderators.

Variable	Mother Sample		Father Sample	
	B	SE	B	SE
Everyday discrimination	0.16 **	0.06	0.13 *	0.05
Maternal closeness	−0.04 *	0.01	-	-
Everyday discrimination x maternal closeness	−0.01	0.01	-	-
Paternal closeness	-	-	−0.03 *	0.01
Everyday discrimination x paternal closeness	-	-	−0.01	0.01
Gender	0.01	0.01	0.02	0.02
Race				
Non-Hispanic Black	0.00	0.03	0.01	0.03
Hispanic	−0.01	0.03	−0.01	0.03
Non-Hispanic Asian	−0.08 *	0.03	−0.08 *	0.04
Age	−0.00	0.00	0.00	0.01
Family structure				
Two parents	0.01	0.02	−0.01	0.02
Single parent	0.03	0.02	0.00	0.02
Other	0.06	0.04	0.07	0.05
Immigrant generation status	0.02	0.04	0.03	0.04
Marital status	−0.06 ***	0.01	−0.05 ***	0.02
Employment status	−0.03	0.01	−0.03	0.01
Household income level				
USD 25,000–49,000	−0.05 *	0.02	−0.08 **	0.03
USD 50,000 or above	−0.09 ***	0.02	−0.13 ***	0.02
Highest educational attainment				
Some college or vocational	−0.01	0.02	−0.01	0.02
Completed college or above	−0.10 ***	0.02	−0.08 ***	0.02
Depression (CES-D) at Wave I	0.16 ***	0.02	0.16 ***	0.02

*—*p* < 0.05; **—*p* < 0.01; ***—*p* < 0.001. Note—all covariates included.

**Table 5:** Summary of OLS regression analysis for everyday discrimination and depression, with satisfaction with communication with mothers and fathers as moderators.

Variable	Mother Sample		Father Sample	
	B	SE	B	SE
Everyday discrimination	0.13 *	0.06	0.09	0.05
Satisfaction with communication with mother	−0.03 *	0.01	-	-
Everyday discrimination x satisfaction with communication with mother	−0.01	0.01	-	-
Satisfaction with communication with father	-	-	−0.03 *	0.01
Everyday discrimination x satisfaction with communication with father	-	-	−0.00	0.01
Gender	0.01	0.01	0.02	0.01
Race				
Non-Hispanic Black	−0.00	0.03	0.01	0.02
Hispanic	−0.01	0.03	−0.01	0.02
Non-Hispanic Asian	−0.07 *	0.03	−0.07 *	0.03
Age	−0.00	0.00	0.00	0.00
Family structure				
Two parents	0.00	0.02	−0.00	0.02
Single parent	0.03	0.02	0.01	0.02
Other	0.06	0.04	0.08	0.05
Immigrant generation status	0.02	0.04	0.03	0.04
Marital status	−0.06 ***	0.02	−0.06 ***	0.02
Employment status	−0.03	0.01	−0.02	0.02
Household income level				
USD 25,000–49,000	−0.05 *	0.03	−0.09 **	0.03
USD 50,000 or above	−0.09 ***	0.02	−0.13 ***	0.02
Highest educational attainment				
Some college or vocational	−0.02	0.02	−0.01	0.02
Completed college or above	−0.10 ***	0.02	−0.09 ***	0.02
Depression (CES-D) at Wave I	0.16 ***	0.02	0.16 ***	0.02

*—*p* < 0.05; **—*p* < 0.01; ***—*p* < 0.001. Note—all covariates included.

## Data Availability

Information on how to access restricted-use Add Health files is available here: https://addhealth.cpc.unc.edu/ (accessed on 25 August 2025).
